# An 11-Year Retrospective Analysis of the Prevalence of Malnutrition Diagnosis at Discharge in a Multi-Site Hospital: The Impact of Introducing a Clinical Nutrition Service

**DOI:** 10.3390/nu17193041

**Published:** 2025-09-24

**Authors:** Giorgia Preatoni, Dario Bertolotti, Giulia Galligani, Nicola Ossola, Massimo Quarenghi

**Affiliations:** 1 Clinical Nutrition and Dietetics, Department of Internal Medicine, Ospedale San Giovanni, Ente Ospedaliero Cantonale (EOC), 6500 Bellinzona, Switzerland; 2 Clinical Nutrition and Dietetics, Department of Internal Medicine, Ospedale La Carità, Ente Ospedaliero Cantonale (EOC), 6600 Locarno, Switzerland; 3 Clinical Nutrition and Dietetics, Department of Internal Medicine, Ospedale di Lugano, Ente Ospedaliero Cantonale (EOC), 6900 Lugano, Switzerland

**Keywords:** malnutrition, NRS, nutrition risk screenig, hospital malnutrition

## Abstract

Background: Nutritional therapy improves prognosis by reducing morbidity and mortality in malnourished hospitalised patients. To determine the occurrence of hospital malnutrition, it is essential to verify healthcare organisations’ ability to identify at-risk patients, considering that malnutrition is often hard to recognise without adequate screening. Methods: The aim of this study is to analyse the temporal evolution in hospitalised patients’ malnutrition, documented as diagnosis discharge letters, over an 11-year period (2014–2024) in four acute care hospitals, with a combined mean of 38,000 inpatients per year. Binomial regression and interrupted time series analysis were used to evaluate temporal evolution in the identification of malnutrition, particularly in relation to the introduction of a nutritional service in 2017. Results: Malnutrition diagnoses increased steadily across hospitals and within internal medicine and surgery departments. Interrupted time series analysis showed a significant rise in diagnostic odds post-intervention, especially in internal medicine. A plateau was observed in the last 3 years, with a mean prevalence of malnutrition of 18.2% for internal medicine (95% CI: 13.6–22.9) and 4.1% for surgery (95% CI: 0.5–7.6). Without the nutritional service, these results likely only would have been reached by 2031 in internal medicine and 2024 in surgery, suggesting an advancement of about eight years in medical wards. Conclusions: The introduction of a nutritional team has probably accelerated and improved the ability to quickly identify and therefore treat malnourished inpatients.

## 1. Introduction

Malnutrition is present in hospitals, but its exact prevalence is difficult to determine because it often depends on the definition or the parameter that has been used, or on how it has been detected [1,2]. Criteria from the Global Leadership Initiative on Malnutrition (GLIM) are often just discussed and not properly used to make hospital malnutrition diagnoses [3]. For the correct codification and retribution of a malnutrition diagnosis, some countries, such as Switzerland, do not use the GLIM criteria but seek a positive result in Nutritional Risk Screening (NRS-2002) and request documented nutritional evaluation and care during hospitalisation [4,5]. The European Society for Clinical Nutrition and Metabolism (ESPEN) guidelines suggest performing malnutrition screening on hospitalised patients [6]. Some Italian regions, such as Lombardy, have made screening a legal obligation but have not suggested which tool to use and how to manage it [7]. Screening is valuable because, as evidenced in the literature, hospitalised malnourished patients have greater morbidity and mortality rate [8]. Nutritional care during hospitalisation has a significant impact on improving prognosis and reducing not only morbidity but also mortality in these patients [9]. Currently, important ongoing studies are evaluating the impact of nutritional care that continues after hospital discharge [10]. It is thereby essential to recognise at-risk or already malnourished patients and to report diagnoses in hospital discharge letters for proper continuation of nutritional therapy during the post-hospitalisation period. Every person involved in the process should know the methods used for screening and how to report a diagnosis. The most efficient screening method is still unclear, which is partly why the process is not easy or smooth. Knowing the malnutrition prevalence is therefore crucial to understand whether a particular care setting can properly detect malnourished patients. It is also important to remember that a positive screening result does not mean that a patient is malnourished but that they are at risk of malnutrition; an assessment is required to confirm or refute the diagnosis. There is often confusion in the literature, with prevalence rates often being reported after screening rather than after actual assessments. Over the past eleven years (01.2014–12.2024) in our multi-site hospital, we conducted a systematic review of malnutrition diagnoses reported in hospital discharge letters. These diagnoses are associated with NRS (Nutritional Risk Screening) and codified through the SwissDRG [4,5]. In the healthcare context, malnutrition is a relevant indicator for clinical outcomes, healthcare costs, and assistance quality [11]. In these eleven years, as predicted, there have been many changes such as the computerisation of nutritional screening, the introduction of a clinical nutrition service (Nutritional Team), and personnel leading various hospital services [12]. We analysed the evolution of malnutrition in detail, evaluating the impact of introducing a clinical nutrition service into the hospital setting.

## 2. Materials and Methods

This is a retrospective, observational, longitudinal, and multicentre study based on administrative data routinely collected by the controlling, coding, and quality services of the four acute hospitals belonging to the Ente Ospedaliero Cantonale (EOC) [13].

### 2.1. History of Evolution of Nutritional Care in Hospitals and Hospital Structures

The Ente Ospedaliero Cantonale (EOC) is the group of public hospitals in the Canton of Ticino, Switzerland [13]. It consists of 4 acute hospitals, 3 smaller local hospitals that do not cover all specialties, and 2 rehabilitation clinics. Over the years, management has become increasingly interdisciplinary. In the past 11 years, excluding rehabilitation clinics, almost 38,000 patients have been admitted annually. In the years before the introduction of the Clinical Nutrition and Dietetic Service (locally a nutritional team in May 2017 in a uniform manner across all locations of the multi-site hospital), a cantonal nutrition commission convened on an annual or bi-annual basis with the objective of providing recommendations to the local commissions present in each hospital on nutrition-related issues, including malnutrition. The composition of the local commissions varied according to the specific characteristics of each hospital facility, but consistently included dietitians and medical practitioners who had a particular interest in nutrition. At that time, Nutritional Risk Screening (NRS), a tool recognised by the European Society for Clinical Nutrition and Metabolism (ESPEN), was used as screening method for malnutrition [3,5,14]. However, a uniform modus operandi for its implementation was not identified, and it was in paper form. In 2015, the Federal Statistical Office clearly set out the parameters required for coding malnutrition in the Medical Coding Manual. These rules have remained largely unchanged since then.

Furthermore, as is still the case today, the nutritional intake of meals was monitored: this is a semi-quantitative monitoring method through the plate system (previously in paper form), where the member of staff collecting the tray estimates the quantity consumed by the patient, reporting in quartiles the data in medical records.

Following the establishment of the cantonal Clinical Nutrition and Dietetic Service, a nutritional team was designated for each hospital to improve the management of malnourished patients and nutritional counselling in general. The local team consists of a medical doctor, who leads it, and dietitians [12].

Before 2017, NRS was carried out on paper, and with the introduction of the Clinical Nutrition and Dietetic Service, it was integrated into computerised and semi-automated medical records. The rest of the process remains unchanged: the first part is carried out by nurses, and the second part by physicians. In practice, the three questions and parameters (weight and height), which were asked/measured when not obtained via the screening tools, have been made mandatory and have become an integral part of the nursing history taken on admission. This device allowed us to systematically perform screening in the first 24–48 h for all patients who were hospitalised. If any of the four items had pathological results, a red alert appeared in the patient’s diagnosis list. The patient’s physician, by activating the alert message, could gain access to the second part of the screening procedure. If the score exceeded three points, the patient was deemed “at risk of malnutrition”, which directly prompted a request for consultation by the Clinical Nutrition and Dietetic Service. The Service also provided training sessions for physicians and nurses in addition to the usual bedside teaching during consultations.

Furthermore, the Service participates regularly in clinical visits in certain high-risk departments, such as visceral surgery, and is also present regularly/daily at the oncology institute. Throughout the year, a variety of internal training courses on the topic of malnutrition are provided.

### 2.2. Data Collection and Analysis

Anonymised data were provided to the managers of the Clinical Nutrition and Dietetic Service in the form of Excel tables. These tables contained the total number of discharged adult patients (aged >16 years) for each year from January 2014 to December 2024, the percentage of patients diagnosed with malnutrition (according to SwissDRG coding), and the absolute number of cases of malnutrition, broken down by department (internal medicine, surgery, and hospital total) [4]. The average number of hospitalisations per year in the study period was approximately 38,000 patients.

All adult inpatient wards for which SwissDRG-coded discharge letters were available were included. It should be noted that not all hospitals have the same wards each year. The data, which were previously aggregated and calculated by the quality services, were reorganised into a long format for statistical analysis.

**Figure 1:**
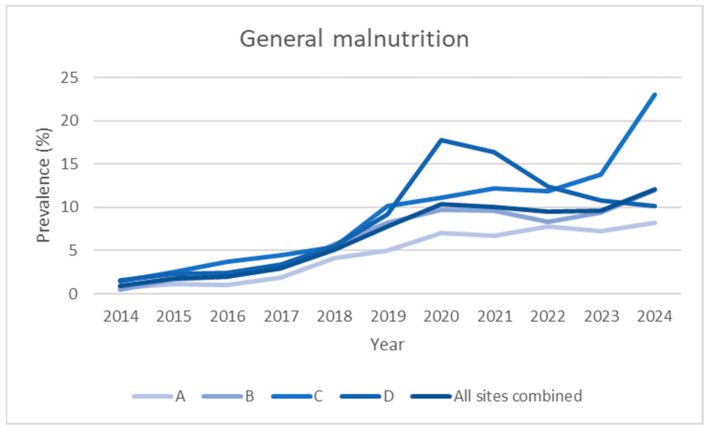
Prevalence data over time (2014–2024), expressed as observed prevalence for individual hospitals and total overall, not divided into departments.

**Figure 2:**
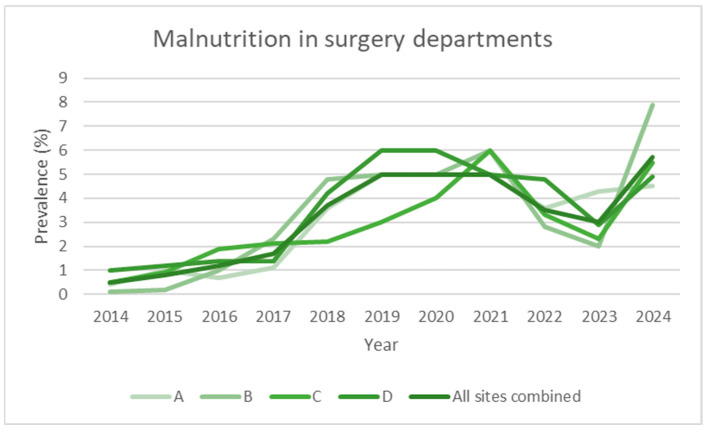
Prevalence data over time (2014–2024), expressed as observed prevalence for individual hospitals and total overall in surgery departments.

**Figure 3:**
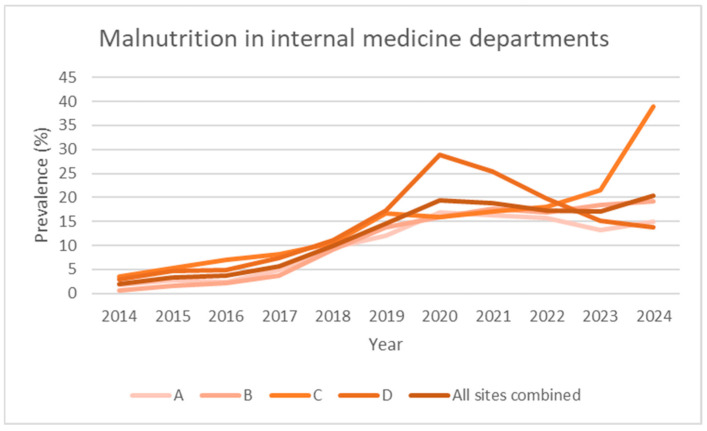
Prevalence data over time (2014–2024), expressed as observed prevalence for individual hospitals and total overall in internal medicine departments.

**Figure 4:**
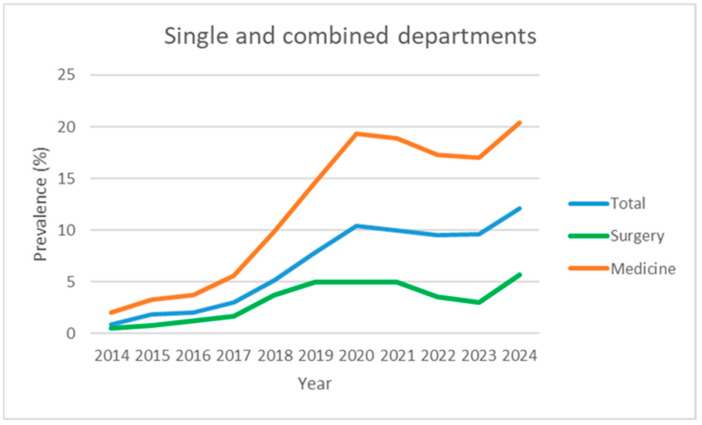
Prevalence data over time (2014–2024), expressed as observed prevalence for single and combined departments, not divided into hospitals.

To facilitate visual interpretation, the graphs were divided by clinical area (internal medicine, surgery, and hospital total) and represent individual hospitals and the aggregate total (Figure 1, Figure 2, Figure 3 and Figure 4). Statistical analyses were conducted using R (version 4.5.1, released 2025-06-13; platform: x86_64-w64-mingw32/x64). Data import and transformation were performed using the readxl, dplyr, and tidyverse packages. Graphical representations were produced using the ggplot2 package. All packages were sourced from CRAN using the Zurich mirror (https://cran.ch.r-project.org, accessed on 14 September 2025), and all code is available in the Supplementary Materials. Generalised linear models (GLMs) with a binomial family and logit link were fitted to estimate the prevalence of malnutrition across clinical domains and hospitals. The variable domain was modelled as a categorical factor with three levels (medicine, surgery, total), with medicine as the reference category, thus providing odds ratios for surgery and total relative to medicine. Each observation corresponded to a hospital–year–domain combination. To account for varying sample sizes, models were fitted using binomial likelihood with the number of malnourished patients and non-malnourished patients (discharges—malnourished) as the response, effectively weighting estimates by the number of discharges.

To assess the potential impact of the COVID-19 pandemic, the binomial GLM was refitted excluding data from the pandemic years (2020–2021). This sensitivity analysis was applied across all hospitals and clinical domains. A separate model restricted to internal medicine patients was also fitted to confirm the robustness of the temporal trend. A *p*-value < 0.05 indicated statistical significance for all tests. To assess the impact of the clinical nutrition service introduced in mid-2017, we conducted a weighted interrupted time series (ITS) analysis using a binomial generalised linear model (GLM) with logit link. The outcome was the proportion of malnourished patients, weighted by discharge volume. The binary indicator “nutrition_service” denoted the presence (1) or absence (0) of the intervention. The analysis covered the years 2015 to 2019, excluding 2017 to avoid transitional bias. An interaction term between year and intervention status was included to estimate both immediate and slope-level changes in diagnostic prevalence across hospital sites and clinical domains.

To formally estimate the post-intervention plateau, segmented regression using restricted cubic splines was implemented via the ns() function from the splines package. Restricted cubic spline models were fitted to annual prevalence data for each domain (medicine, surgery, total) using binomial generalised linear models weighted by discharge volume.

The first derivative of the fitted spline curve was computed numerically to assess the rate of change in prevalence over time. Derivative calculations were performed using base R functions, with the purrr and tibble packages used to structure domain-specific outputs. This approach enabled formal identification of the stabilisation phase, defined as the point at which the derivative approached zero.

To estimate the prevalence plateau, the mean of the most recent three available years (2022–2024) was calculated, with 95% confidence intervals obtained via the standard error of the mean (SEM) and Student’s t-distribution.

The study was conducted in accordance with the Declaration of Helsinki and approved by the hospital’s quality control department. It is exempt from Swiss ethics committee authorisation as it was based exclusively on irreversibly anonymous secondary data.

## 3. Results

Table 1 and Figure 1 and Figure 4 show the evolution of the prevalence (in percentage) of the diagnosis of malnutrition according to SwissDRG coding, recorded from 2014 (the year in which systematic collection began) to the end of 2024, for a total of 11 years.

The data are broken down based on the four acute care hospitals that comprise the multi-site hospital, and the aggregate (all sites combined) value is shown in the last row of each section. Over the 11 years of observation, the number of inpatients remained stable, averaging approximately 38,000 admissions per year.

The first part of Table 1 (section A) shows data for all departments in each hospital, including internal medicine and surgery, as well as urology, geriatrics, haemato-oncology, and gynaecology. It should be noted that not all hospitals have the same specialised departments on a continuous basis.

Over the 2014–2024 period, the prevalence of malnutrition increased steadily across all areas analysed: it increased from 2.0% to 20.4% in internal medicine, from 0.5% to 5.7% in surgery, and from 0.9% to 12.1% in the hospital total (all specialties) (Figure 1, Figure 2, Figure 3 and Figure 4). This increase occurred consistently across all four hospitals.

The binomial GLM demonstrated a consistent annual increase in the odds of malnutrition diagnosis across clinical domains, with an estimated rise of approximately 23% per year (OR = 1.23, 95% CI: 1.22–1.24). Using internal medicine as the reference category, the surgical domain showed markedly lower odds of malnutrition diagnosis (OR = 0.23, 95% CI: 0.22–0.24), while the hospital-wide total exhibited an intermediate level (OR = 0.52, 95% CI: 0.50–0.53). Among hospitals, Hospital C displayed the highest odds ratio (OR = 1.84, 95% CI: 1.79–1.89), which prompted further investigation. Given its designation as a regional COVID-19 referral centre in 2020–2021, a separate model was fitted excluding those years to assess potential pandemic-related amplification.

To evaluate the potential impact of the COVID-19 pandemic, the model was refitted excluding data from the pandemic years (2020–2021). The annual increase in odds remained stable (OR = 1.23, 95% CI: 1.23–1.24), indicating the robustness of the underlying temporal trend. The effect associated with Hospital C, designated as a regional COVID-19 referral centre, remained elevated following exclusion of the pandemic years (OR = 1.98 vs. 1.83), suggesting a structural difference rather than a transient pandemic-related amplification. Full model estimates, including odds ratios and confidence intervals, are presented in Supplementary Tables S1 and S2.

**Figure 5:**
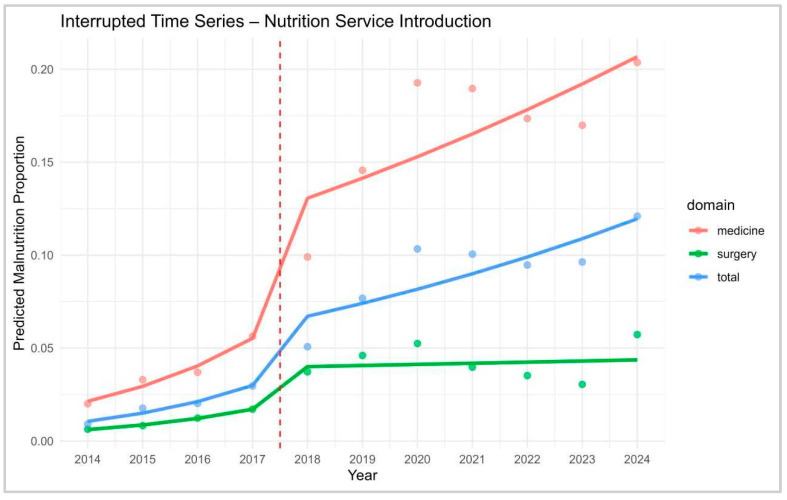
ITS modelling restricted to post-intervention period. Interrupted Time Series (ITS) analysis of predicted malnutrition prevalence across domains (medicine, surgery, total). The vertical dashed line indicates the introduction of the Nutrition Service in 2017.

To assess the impact of the clinical nutrition service introduced in mid-2017, a weighted interrupted time series (ITS) analysis was conducted across internal medicine, surgery, and the total hospital population (Figure 5). The analysis covered the years 2015–2019, excluding 2017 as a transition year to avoid implementation bias. This approach allowed for the estimation of both immediate (level) and longitudinal (slope) changes in diagnostic prevalence, weighted by discharge volume. In internal medicine departments, the model revealed a significant immediate increase in the odds of malnutrition diagnosis following the intervention (OR = 4.12, 95% CI: 3.78–4.49, *p* < 0.001), accompanied by a significant attenuation in the post-intervention slope (OR = 0.79, 95% CI: 0.75–0.83, *p* < 0.001). This suggests a rapid uptake followed by a stabilisation phase, possibly reflecting saturation in identification efforts. In surgical departments, the immediate effect was also significant though less pronounced (OR = 2.31, 95% CI: 2.12–2.52, *p* < 0.001), with a post-intervention slope reduction (OR = 0.84, 95% CI: 0.80–0.88, *p* < 0.001), indicating a similar but more gradual pattern. For the total hospital population, the ITS model showed a marked level change (OR = 3.43, 95% CI: 3.27–3.60, *p* < 0.001) and a significant deceleration in the trend (OR = 0.785, 95% CI: 0.751–0.820, *p* < 0.001), confirming the overall effectiveness of the intervention (Supplementary Tables S3 and S4).

**Figure 6:**
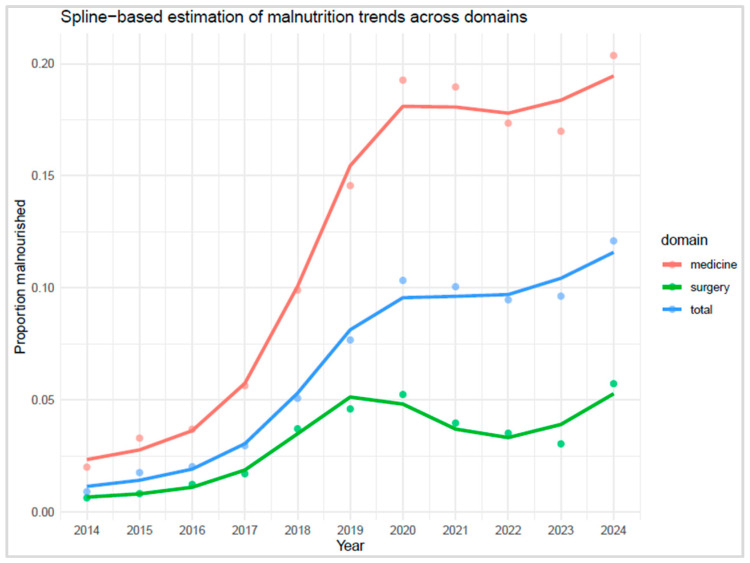
Spline-based estimation of malnutrition trends across domains.

Spline modelling revealed a marked increase in malnutrition prevalence across all domains until approximately 2020, followed by a clear deceleration in growth. While spline curves were fitted across the full time series for visual continuity, formal ITS modelling was restricted to the post-intervention segment to estimate the plateau phase (Figure 6). The first derivative of the fitted spline curves approached zero in 2021 for both internal medicine and the hospital total, indicating a stabilisation phase. Specifically, the derivative in internal medicine was −0.0003 in 2021 and −0.0028 in 2022, suggesting curve flattening. In the hospital total, the derivative was +0.0006 in 2021 and +0.0008 in 2022, consistent with a plateau. In contrast, the surgery domain showed a transient decline in 2021 (−0.0111), followed by continued variability, with derivatives of −0.0038 in 2022 and +0.0059 in 2023, indicating no sustained stabilisation (Supplementary Tables S5 and S6).

Given the observed stabilisation, the average of the last three years (2022–2024) was retained as a pragmatic estimate of plateau prevalence: 18.2% in internal medicine (95% CI: 13.6–22.9), 4.1% in surgery (95% CI: 0.5–7.6), 10.4% in the hospital total (95% CI: 6.7–14.1). Regarding pre-intervention trends, it is estimated that these levels would have only been reached around 2031 for internal medicine and the hospital total. Surgery, however, would have reached comparable stabilised prevalence around 2024. This suggests a significant acceleration (approximately 7–8 years) in medical departments, presumably attributable to the consolidation and spread of systematic reporting by the nutrition team. To estimate a plateau following the introduction of the service, the average of the last three years (2022–2024) was calculated: the plateau was 18.2% in internal medicine (95% CI: 13.6–22.9), 4.1% in surgery (95% CI: 0.5–7.6), and 10.4% in the hospital total (95% CI: 6.7–14.1). Regarding pre-intervention trends, it is estimated that these levels would have only been reached around 2031 for internal medicine and for the general hospital total. Surgery, however, would have still reached the plateau around 2024. This suggests a significant advance (approximately 7–8 years) in medical departments, presumably attributable to the consolidation and spread of systematic reporting by the nutrition team.

## 4. Discussion

Recent studies have provided substantial scientific evidence that the proactive management of patients at risk of malnutrition or those who are malnourished in a hospital setting can lead to a favourable prognosis [9]. The intervention has been demonstrated to reduce the hospital length of stay while lowering the risk of infection and mortality [9]. Nutritional therapy has also been demonstrated to be a cost-effective intervention [15]. The clinical studies presenting these findings identified patients at risk of malnutrition or those who are malnourished through a well-codified and protocoled procedure. In the absence of screening, a significant proportion of malnourished patients, particularly those of normal weight or those with sarcopenic obesity (for which a definition and diagnostic criteria were established in 2022 according to the ESPEN and EASO Consensus Statement), may not be identified, resulting in suboptimal care being provided [16]. Among the factors that may have led to this increase in diagnoses, in addition to the implementation of nutritional screening, there may also be the ageing of the population (malnutrition is in fact more prevalent in people over 65) and a deterioration in socio-economic status, resulting in food insecurity, accounts for 7% of malnutrition cases in Switzerland) [17,18].

One study demonstrated that nutritional therapy had an impressive impact, resulting in a reduction in morbidity and mortality in malnourished patients; this study involved approximately 2000 randomised, multi-site hospital patients systematically detected with NRS tools [9]. Therefore, to obtain and reproduce these results in hospitalised patients, it is essential to identify those who are at risk of malnourishment. As detecting malnourished patients can be challenging, the guidelines propose implementing a structured screening process [6]. Certain regions, such as Lombardy in Italy, have imposed a legal obligation for healthcare facilities to introduce screening, with economic penalties for hospitals that do not perform it [7]. However, there are no guidelines on how to conduct screening, including which tool to use, who should perform it on the patient, how quickly, in what form, etc., so it is difficult to monitor its use. Future research should evaluate which screening processes achieve better detection of malnourished patients in hospital settings and not just conduct a comparison between various tools, as other studies have done [19,20]. We observed in our work that the introduction of a dedicated team accelerated expected achievements, especially for internal medicine departments.

Having a solid, reliable, and up-to-date literature on the expected prevalence in a particular region is essential to periodically verifying the quality of work. A previous study of Khalatbari-Soltani et al. highlighted that the prevalence of reported undernutrition in Switzerland had increased from 0.32% in 1998 to 3.97% in 2014, with significant differences depending on the canton (from 0.18% to 2.13% in Ticino, with documented treatment increasing from 0% to 32.9%) [21].

Introducing a clinical nutrition service or a nutritional team changes the way patients are cared for. In a previous study [12], our team had already described a probable different therapeutic approach: in the years following the introduction of the nutritional team, we reported a reduction in the use of artificial nutrition, especially parenteral nutrition, in favour of an increase in oral supplements (less invasive therapy). This is probably a sign of more individualised and less standardised nutritional care. Clinical nutrition, as a specialty in its own right, is quite young; for example, in Switzerland, it has only been recognised for a few years [22]. A review with a meta-analysis conducted by Gomes et al. clearly demonstrated the favourable impact of nutritional management in malnourished patients, which has been covered more in recent studies compared to older ones [23].

In our study, we observed a plateau of around 18.2% (95% CI: 13.6–22.9) in the prevalence of malnutrition in the internal medicine departments of the four hospitals over the past 3 years. This value reflects that reported in a previous multi-centre study conducted in the same country [24]. We can therefore assume that the general internal medicine departments of European hospitals have a minimum rate of 18% of malnourished patients. The pre-intervention linear growth curves project that these levels would likely have only been reached by around 2031 for internal medicine, demonstrating that the introduction of a nutritional team accelerated and improved patient detection. It is reasonable to hypothesise that the preparations for the introduction of the service, which had been underway for two or three years, were the cause of the statistically insignificant results.

Regarding the surgical departments, we observed a plateau of around 4.1% (95% CI: 0.5–7.6) in the prevalence of malnutrition, a value that has remained stable over the past three years (2022–2024). The screening process is identical in modality to that applied for internal medicine. This finding was consistently observed in all four hospitals involved, indicating a consolidated trend. This value is lower than those reported in the international literature, where the prevalence of surgical malnutrition is often cited between 10% and 50% depending on various settings [25,26,27]. A possible explanation could be linked to the type of patients present in our departments: surgical units include both elective and urgent cases, with a heterogeneous population in terms of age (including young adults) and conditions (first admission vs. readmission), which could dilute the data by including more patients not at risk. Indeed, the present study does not report on the prevalence of positive screenings; rather, it focuses on the diagnoses that are detected through SwissDRG coding. Moreover, the available literature frequently restricts the study to a specific surgical population, namely visceral, oncological, and trauma surgery. This observation represents a critical point to be explored in future studies since it is well known that malnutrition, even in the surgical setting, is associated with an increase in postoperative complications, hospital stays, and overall morbidity [28]. Another hypothesis is linked to the fact that over the past three to four years, our visceral surgery service has implemented prehabilitation protocols for all patients diagnosed with cancers affecting the pancreas, liver, stomach, and oesophagus. Following a diagnosis, patients are expeditiously referred to the clinical nutrition service to undergo a comprehensive prehabilitation programme encompassing both nutritional and physical components. This approach is predicated on the premise that by the time of surgery, patients will be stabilised and no longer be malnourished, as already demonstrated in literature [29]. The underestimation of malnutrition in surgery may also be indicative of a certain cultural and clinical inertia, in contrast to the observations made in internal medicine departments, where the nutritional approach is generally more integrated and proactive.

The data also brought to light the period of the novel coronavirus (SARS-CoV-2) pandemic; it was quickly analysed given that the anomaly in the data on the internal medicine service of a hospital (D) immediately emerged (Table 1). Within the internal medicine service of the hospital that was converted into a COVID-19 centre for patients in the entire region during the initial phase of the pandemic, the predominance of malnutrition suggests (although this remains to be substantiated) that malnourished patients were profoundly impacted by the infection or experienced malnutrition during protracted periods of hospitalisation [30,31]. The initial phase of the epidemic was characterised by a wide spectrum of gastrointestinal symptoms, ranging from sensory alterations such as changes in taste and smell to more severe manifestations including loss of appetite and diarrhoea. A significant proportion of cases also required orotracheal intubation, which could persist for extended periods, often spanning days or weeks, resulting in protracted recovery times [32]. Excluding the data from these two years, the increase appears more homogeneous. However, the impact of the introduction of the nutritional team is not statistically significant between the pre- and post-pandemic periods.

From an organisational perspective, as outlined in the previous section, the functioning of our hospitals was previously characterised by independence. A commonality across studies was the monitoring of patient intake during the three main meals (a practice that remains in place), accomplished through the documentation of the patients’ food consumption on a schematic plate divided into four segments (semi-quantitative estimate). This responsibility is typically assigned to either the nurse or the nursing assistant. Interpreting this monitoring process is challenging as it does not differentiate between the specific nutrients consumed, namely protein, carbohydrates, and fibre. Furthermore, it is imperative to ascertain the quantity of food received by the patient, namely a half portion or a full portion. For instance, half a plate of a full portion is reported as being equivalent to the intake of half a plate of a half portion (approximately 800 kcal vs. 1700, by our standards). A diploma thesis published as a poster highlighted that two different nutritional monitoring methods (the first consisting solely of semi-quantitative monitoring of nutritional intake, as explained above, and the second involving both the former and the completion of NRS in paper format) did not allow for the detection of malnutrition in our hospitals, which was reported as a diagnosis in the discharge letter. The prevalence was higher than 2% in the medical department where the second method (nutritional intake monitoring and NRS) was used, but the paper screening sheet was often missing, in addition to other problems [33]. The accurate diagnosis of malnutrition is of paramount importance as it signifies that the patient was adequately evaluated. This approach facilitates enhanced modification of the DRG while ensuring the continuity of established nutritional therapy, acknowledging the often prolonged response time to malnutrition. Over the years, a fully computerised system has been implemented, which has changed its application. Despite the general awareness of its significance, deficits in its compilation are still frequently observed (in informal and ongoing audits), with estimates including one-third of screenings not being performed by the nurse (the first part of the NRS), one-third not being performed by the physician (despite having an alert), and one-third of screenings being lost due to general causes, such as screening performed but diagnosis not reported. It is imperative to record the diagnosis in writing so that the problem can be reported to the post-hospitalisation team. The EFFORT II study is currently underway, with a primary focus on this aspect [10].

It is important to consider that there are limitations to the study, which should be taken into account when interpreting the results: given the retrospective nature of our work, the study enables only the observation of the phenomenon’s evolution over time and the formulation of hypotheses regarding the events that transpired. A further significant limitation is that the data are highly dependent on the quality of the International Classification of Diseases (ICD)/SwissDRG coding [4]. As previously mentioned, SwissDRG coding for malnutrition was introduced in 2015 and has remained largely unchanged since then; consequently, data from 2014 to 2015 might be impacted by this factor. Moreover, the coding is not centralised but is linked to individual hospitals and thus varies. However, the large numbers and years of observation allow this limitation to be mitigated [4]. Another aspect is that NRS screening became computerised in 2017, the year in which the Clinical Nutrition and Dietetic Service was introduced, so this could have an impact on the data relating to previous years. In addition, the potential for unrecorded confounding factors, such as case mix or clinical severity, cannot be disregarded. Moreover, given that this is a retrospective study, it is not possible to exclude other fluctuations, such as those related to changes in the head of a service or broader policies.

In conclusion, the diagnosis that has been reported is consistent with NRS, which has not yet been standardised with the increasingly desirable GLIM criteria. Specifically, the GLIM criteria include phenotypic factors, such as non-volitional weight loss, low BMI and reduced muscle mass, as well as aetiological factors, such as reduced food intake or assimilation and disease burden/inflammation. The NRS screening criteria involve calculating a score based on age, BMI, involuntary weight loss and disease severity, with points assigned for each criterion. Patients with an overall score of three or more are considered to be at nutritional risk. Nevertheless, in the absence of a screening process or a structured method for detecting malnutrition, the GLIM criteria alone are insufficient for identifying all malnourished patients admitted to a hospital.

The present study demonstrates the efficacy of systematic and continuous data collection over time as a means of comparing facilities and departments. This approach facilitates the monitoring of the identification of malnourished patients against clinical and epidemiological expectations. This study also shows that incorporating a nutritional team facilitates the identification of patients at risk, thereby enabling the provision of adequate care. However, it should be noted that some variations have been observed between different departments. From this standpoint, it is imperative for our group to undertake a further investigation into the observed discrepancies between medicine and surgery. This investigation should also encompass the potential for instigating organisational modifications to surgical pathways with the objective of enhancing alignment with the Enhanced Recovery After Surgery (ERAS) recommendations [34]. Future studies should focus on identifying the exact prevalence of malnutrition in various surgical settings and which screening/assessment path is best for the surgical setting.

## 5. Conclusions

Malnutrition is common in hospitals, but its prevalence depends on both the department and the method of detection. Some services, such as internal medicine, appear more inclined to incorporate structured protocols for detecting malnutrition into their workflow than services such as surgery. Although this is a retrospective study for which no adjustments were made for case mix or policy changes, the results show that introducing a nutritional team allows the expected prevalence plateau for a given service to be reached sooner, improving the identification of malnourished patients and, consequently, the number of patients cared for.

## Figures and Tables

**Table 1:** The evolution of the prevalence (in percentage terms) of malnutrition diagnoses (2014–2024) broken down by hospitals and departments.

**A. General Malnutrition**											
hospital	2014	2015	2016	2017	2018	2019	2020	2021	2022	2023	2024
A	0.7	1.1	1.0	1.9	4.1	5.0	7.0	6.7	7.8	7.2	8.2
B	0.5	1.7	2.0	3.0	5.8	8.2	9.7	9.6	8.3	9.4	12.0
C	1.5	2.5	3.7	4.5	5.4	10.2	11.1	12.2	11.9	13.8	23.1
D	1.6	2.3	2.4	3.4	5.5	9.2	17.8	16.4	12.4	10.8	10.2
All sites combined	0.9	1.8	2.0	3.0	5.1	7.8	10.4	10.0	9.5	9.6	12.1
**B. Malnutrition in Surgery Departments**											
hospital	2014	2015	2016	2017	2018	2019	2020	2021	2022	2023	2024
A	0.4	1.0	0.7	1.1	3.6	5.0	5.0	5.0	3.6	4.3	4.5
B	0.1	0.2	1.0	2.3	4.8	5.0	5.0	6.0	2.8	2.0	7.9
C	0.5	0.9	1.9	2.1	2.2	3.0	4.0	6.0	3.3	2.3	5.5
D	1.0	1.2	1.4	1.4	4.2	6.0	6.0	5.0	4.8	2.9	4.9
All sites combined	0.5	0.8	1.2	1.7	3.7	5.0	5.0	5.0	3.5	3.0	5.7
**C. Malnutrition in Internal Medicine Departments**											
hospital	2014	2015	2016	2017	2018	2019	2020	2021	2022	2023	2024
A	1.7	2.8	2.4	4.8	9.6	12.0	16.8	16.2	15.7	13.2	15.0
B	0.7	1.6	2.2	3.8	9.1	13.8	15.9	17.6	16.9	18.5	19.2
C	3.6	5.3	7.1	8.1	10.4	16.6	16.0	17.1	18.1	21.6	38.9
D	2.9	4.6	4.9	7.3	11.0	17.3	28.9	25.3	19.7	15.2	13.7
All sites combined	2.0	3.3	3.7	5.6	9.9	14.6	19.3	18.9	17.3	17.0	20.4

## Data Availability

The data presented in this study are available on request from the corresponding author. The data are not publicly available due to institutional policy and competitive restrictions. However, anonymised CSV files and the full R analysis script have been provided to reviewers for validation purposes.

## Supplementary Materials

The following supporting information can be downloaded at: https://www.mdpi.com/article/10.3390/nu17193041/s1. Table S1: Binomial GLM (weighted by discharges)—All years. Table S2: Binomial GLM (weighted by discharges)—No-COVID years (excl. 2020–2021). Table S3: Interrupted Time Series (ITS) Model—Nutrition Service (No Interaction). Table S4: Interrupted Time Series (ITS) Model—Nutrition Service (With Interaction). Table S5: Spline Regression Model for Malnutrition Proportion—Nutrition Service (Trend and Plateau Estimation). Table S6: First Derivative of Spline Regression—Nutrition Service (Rate of Change in Malnutrition Proportion).

## Abbreviations

The following abbreviations are used in this manuscript:

EOC	Ente Ospedaliero Cantonale
GLIMs	Global Leadership Initiative on Malnutrition
NRS	Nutritional Risk Screening
ICD	International Classification of Diseases
