# Supplementary material for: An 11-Year Retrospective Analysis of the Prevalence of Malnutrition Diagnosis at Discharge in a Multi-Site Hospital: The Impact of Introducing a Clinical Nutrition Service
Source: Nutrients. 2025 Sep 24;17(19):3041. doi: 10.3390/nu17193041 (PMC12525654; doi:10.3390/nu17193041)
Supplement: Supplementary file 1 [file nutrients-17-03041-s001.zip › nutrients-3857767-supplementary.pdf]

1. **Supplementary Table S1.**

Binomial GLM (weighted by discharges) — All years

term	OR	95% CI low	95% CI high	p-value
Year (per 1-year increase)	1.2234339	1.2191749	1.2277197	<0.001
Surgery vs Medicine	0.2190516	0.2086603	0.2298384	<0.001
Total vs Medicine	0.5141337	0.5031502	0.5253708	<0.001
Hospital B vs A	1.2755823	1.2415152	1.3106131	<0.001
Hospital C vs A	1.8264297	1.7725967	1.8818704	<0.001
Hospital D vs A	1.6603323	1.6127907	1.7092699	<0.001

Odds ratios (OR) with 95% confidence intervals (CI) from a binomial GLM with logit link, using the proportion of malnourished patients as the response and discharges as weights. Reference levels: domain = medicine; hospital = A. Year modeled continuously.

2. **Supplementary Table S2.**

Binomial GLM (weighted by discharges) — No-COVID years (excl. 2020–2021)

term	OR	95% CI low	95% CI high	p-value
Year (per 1-year increase)	1.2288867	1.2243628	1.2334418	<0.001
Surgery vs Medicine	0.2260891	0.2137431	0.2389805	<0.001
Total vs Medicine	0.5185498	0.5055943	0.5318572	<0.001
Hospital B vs A	1.2798552	1.2399547	1.3210781	<0.001
Hospital C vs A	1.9762751	1.9096916	2.0451657	<0.001
Hospital D vs A	1.4656425	1.4157904	1.5172200	<0.001

Same model as in table S1, but above but excluding data from 2020–2021 excluded to assess potential pandemic-related amplification. The response variable remains the proportion of malnourished patients, weighted by discharges. Reference levels unchanged: domain = medicine; hospital = A.

3. Supplementary Table S3.

Interrupted Time Series (ITS) Model – Nutrition Service (No Interaction)

term	estimate	std.error	statistic	p.value	conf.low	conf.high
(Intercept)	3.23E-90	5.349835	-38.5168	0	9E-95	1.15E-85
year	1.105601	0.002655	37.81122	0	1.099865	1.111372
hospitalB	1.273752	0.013813	17.51732	1.06E-68	1.239744	1.308722
hospitalC	1.830359	0.015264	39.60304	0	1.776396	1.885933
hospitalD	1.666597	0.014828	34.44798	4.8E-260	1.618857	1.71574
domainsurgery	0.223772	0.024655	-60.7228	0	0.213158	0.234789
domaintotal	0.516115	0.011027	-59.9797	0	0.505086	0.527399
nutrition_service	3.430686	0.024828	49.65142	0	3.267987	3.602044

Binomial GLM with logit link. Outcome: proportion of malnourished patients, weighted by discharge volume. The year 2017 was excluded to avoid transitional bias.

4. Supplementary Table S4.

Interrupted Time Series (ITS) Model – Nutrition Service (With Interaction)

term	estimate	std.error	statistic	p.value	conf.low	conf.high
(Intercept)	5.8E-299	45.18994	-15,196	3.76E-52	0	1.4E-260
year	1.403414	0.022424	15.11337	1.32E-51	1.343202	1.466627
nutrition_service	2.2E+212	45.51106	10.74325	6.38E-27	4.9E+173	1.5E+251
hospitalB	1.273603	0.013812	17.50997	1.2E-68	1.239601	1.308567
hospitalC	1.829796	0.015263	39.58553	0	1.775854	1.885349
hospitalD	1.666461	0.014827	34.4441	5.5E-260	1.618727	1.715598
domainsurgery	0.223981	0.024654	-60.6884	0	0.213358	0.235008
domaintotal	0.516273	0.011027	-59.957	0	0.505241	0.527559
year:nutrition_service	0.785049	0.022583	-10.7165	8.51E-27	0.750981	0.820497

Binomial GLM with logit link. Outcome: proportion of malnourished patients, weighted by discharge volume. The year 2017 was excluded to avoid transitional bias. The model includes an interaction term between year and intervention status.

5. Supplementary Table S5.

Spline Regression Model for Malnutrition Proportion – Nutrition Service (Trend and Plateau Estimation)

domain	year	discharges	malnourished	prop	predicted
medicine	2014			0.020052887	0.023425700

domain	year	discharges	malnourished	prop	predicted
medicine	2015			0.032996922	0.027739433
medicine	2016			0.036966825	0.036317169
medicine	20017			0.056364974	0.057435258
medicine	2018			0.099029702	0.100686379
medicine	2019			0.145608058	0.154500137
medicine	2020			0.192673930	0.180982722
medicine	2021			0.189606357	0.180669308
medicine	2022			0.173471061	0.177923223
medicine	2023			0.169853675	0.183732425
medicine	2024			0.203645613	0.194520321
surgery	2014			0.006330197	0.006677125
surgery	2015			0.008244994	0.008118010
surgery	2016			0.012337662	0.011066795
surgery	2017			0.017097061	0.018750573
surgery	2018			0.037155189	0.034947784
surgery	2019			0.046006158	0.051311744
surgery	2020			0.052436310	0.048161930
surgery	2021			0.039706517	0.037015467

domain	year	discharges	malnourished	prop	predicted
surgery	2022			0.035185702	0.033219787
surgery	2023			0.030434783	0.039084357
surgery	2024			0.057251372	0.052769347
total	2014			0.009095842	0.011455960
total	2015			0.017622258	0.014190925
total	2016			0.020255809	0.019163669
total	2017			0.029617649	0.030493029
total	2018			0.050748926	0.053049742
total	2019			0.076763751	0.081323140
total	2020			0.103335708	0.095618601
total	2021			0.100531119	0.096238070
total	2022			0.094676165	0.097023304
total	2023			0.096314930	0.104289962
total	2024			0.120953142	0.115858543

Spline regression models with 4 degrees of freedom were applied to estimate the temporal trend in malnutrition proportion across clinical domains. Predicted values reflect smoothed estimates accounting for discharge volume, allowing for potential plateau detection.

6. Supplementary Table S6.

First Derivative of Spline Regression – Nutrition Service (Rate of Change in Malnutrition Proportion)

domain	year	discharges	malnourished	prop	predicted	deriv
medicine	2014			0.020052887	0.023425700	
medicine	2015			0.032996922	0.027739433	0.0043137325
medicine	20016			0.036966825	0.036317169	0.0085777362
medicine	2017			0.056364974	0.057435258	0.0211180890
medicine	2018			0.099029702	0.100686379	0.0432511213
medicine	2019			0.145608058	0.154500137	0.0538137574
medicine	2020			0.192673930	0.180982722	0.0264825853
medicine	2021			0.189606357	0.180669308	-0.0003134139
medicine	2022			0.173471061	0.177923223	-0.0027460852
medicine	2023			0.169853675	0.183732425	0.0058092025
medicine	2024			0.203645613	0.194520321	0.0107878961
surgery	2014			0.006330197	0.006677125	
surgery	2015			0.008244994	0.008118010	0.0014408851
surgery	2016			0.012337662	0.011066795	0.0029487857
surgery	2017			0.017097061	0.018750573	0.0076837779

domain	year	discharges	malnourished	prop	predicted	deriv
surgery	2018			0.037155189	0.034947784	0.0161972104
surgery	2019			0.046006158	0.051311744	0.0163639602
surgery	2020			0.052436310	0.048161930	-0.0031498135
surgery	2021			0.039706517	0.037015467	-0.0111464634
surgery	2022			0.035185702	0.033219787	-0.0037956797
surgery	2023			0.030434783	0.039084357	0.0058645695
surgery	2024			0.057251372	0.052769347	0.0136849901
total	2014			0.009095842	0.011455960	
total	2015			0.017622258	0.014190925	0.0027349655
total	2016			0.020255809	0.019163669	0.0049727438
total	2017			0.029617649	0.030493029	0.0113293599
total	2018			0.050748926	0.053049742	0.0225567126
total	2019			0.076763751	0.081323140	0.0282733984
total	2020			0.103335708	0.095618601	0.0142954604
total	2021			0.100531119	0.096238070	0.0006194695
total	2022			0.094676165	0.097023304	0.0007852344

domain	year	discharges	malnourished	prop	predicted	deriv
total	2023			0.096314930	0.104289962	0.0072666571
total	2024			0.120953142	0.115858543	0.0115685812

This table reports the year-on-year change in the predicted proportion of malnourished patients across clinical domains (medicine, surgery, total), estimated using spline regression models with 4 degrees of freedom. The first derivative quantifies the rate of change in malnutrition prevalence over time, allowing for the identification of inflection points and potential plateaus. Derivative values are not available for the initial year (2014) due to lack of preceding data.
